# People’s perspectives and expectations on preparedness against earthquakes: Tehran case study

**DOI:** 10.5249/jivr.v2i2.25

**Published:** 2010-06

**Authors:** Katayoun Jahangiri, Yasamin O Izadkhah, Ali Montazeri, Mahmood Hosseini

**Affiliations:** ^*a*^Health Services Management Department, Iranian Institute for Health Sciences Research (IHSR), Iranian Academic Center for Education, Culture and Research (ACECR).; ^*b*^Risk Management Research Centre, International Institute of Earthquake Engineering and Seismology, (IIEES).; ^*c*^Center of Excellence on Risk Management, International Institute of Earthquake Engineering and Seismology, (IIEES).

## Abstract

**Background:**

Public education is one of the most important elements of earthquake preparedness. The present study identifies methods and appropriate strategies for public awareness and education on preparedness for earthquakes based on people's opinions in the city of Tehran.

**Methods:**

This was a cross-sectional study and a door-to-door survey of residents from 22 municipal districts in Tehran, the capital city of Iran. It involved a total of 1 211 individuals aged 15 and above. People were asked about different methods of public information and education, as well as the type of information needed for earthquake preparedness.

**Results:**

"Enforcing the building contractors' compliance with the construction codes and regulations" was ranked as the first priority by 33.4% of the respondents. Over 70% of the participants (71.7%) regarded TV as the most appropriate means of media communication to prepare people for an earthquake. This was followed by "radio" which was selected by 51.6% of respondents. Slightly over 95% of the respondents believed that there would soon be an earthquake in the country, and 80% reported that they obtained this information from "the general public". Seventy percent of the study population felt that news of an earthquake should be communicated through the media. However, over fifty (58%) of the participants believed that governmental officials and agencies are best qualified to disseminate information about the risk of an imminent earthquake. Just over half (50.8%) of the respondents argued that the authorities do not usually provide enough information to people about earthquakes and the probability of their occurrence. Besides seismologists, respondents thought astronauts (32%), fortunetellers (32.3%), religious figures (34%), meteorologists (23%), and paleontologists (2%) can correctly predict the occurrence of an earthquake. Furthermore, 88.6% listed aid centers, mosques, newspapers and TV as the most important sources of information during the aftermath of an earthquake, Discussion: A participatory approach to earthquake-preparedness planning is recommended. This would ensure that program planners use methods, tools, media, and educational materials that are compatible with the culture, needs, and skills of the local communities.

**Conclusions:**

The findings of this study also reveal methods and tools that the local community considers to be most effective for earthquake-preparedness planning and management. The development of an earthquake-resistance and a safe community requires a high level of collaboration between broadcasting organizations, seismologists, experts in the disaster- preparedness field, as well as the local community. This will allow for timely planning, development, and dissemination of essential information to all stakeholders including the local communities.

## Introduction

 In a recent World Disaster Report, Iran was cited among the countries which have suffered the highest number of casualties from earthquakes.^1^The country is located in the Alpine-Himalayan seismic belt, which is one of the most active tectonic regions of the world, making it vulnerable to natural disasters such as earthquakes. Indeed, in the past few centuries, Iran has endured quite a few large and destructive earthquakes.

**Figure1:**
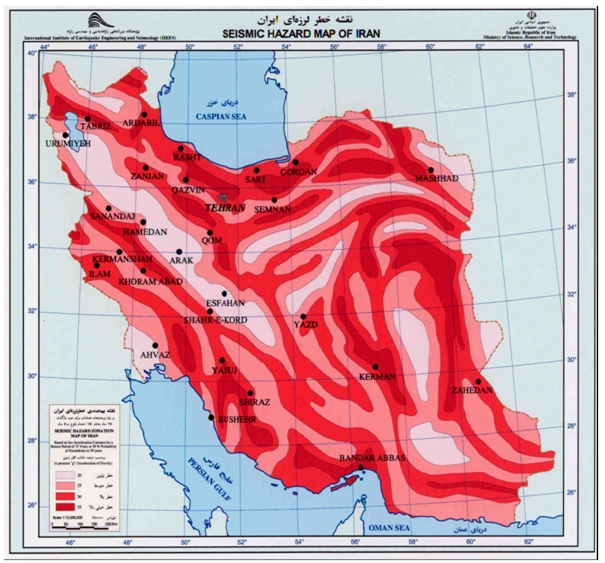
			Figure 1. Seismic Hazard Map of Iran. Reproduced from International Institute of Earthquake Engineering and Seismology (IIEES) Website (www.iiees.ac.ir) with permission from IIEES.

**Figure2:**
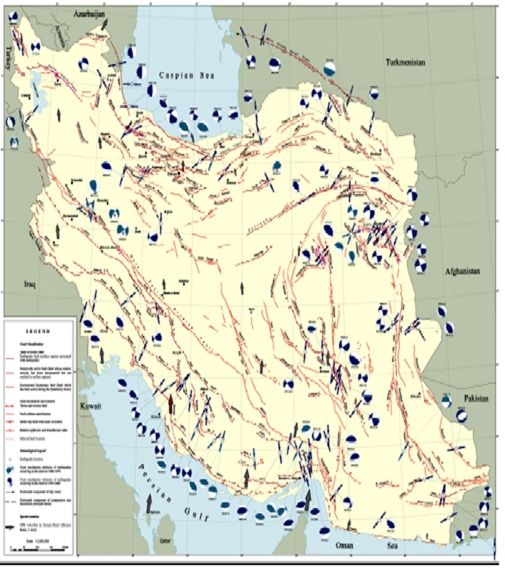
			Figure 2. Major Active Faults of Iran. Reproduced from International Institute of Earthquake Engineering and Seismology (IIEES) Website (www.iiees.ac.ir) with permission from IIEES.

Figures (1)and Figures (2) illustrate that more than 70% of the large cities in Iran are located in the vicinity of seismic faults and in some cases, the active faults pass through these cities, increasing the risk of loss of life.

Worldwide, evidence shows that there is an upward trend       in the number of people who are impacted physically, socially, and economically by the threats of natural disasters.^2^ Although a society is an amalgamation of various stakeholders, when a natural disaster strikes, three groups of stakeholders are most impacted: the authorities, the experts, and the general public (i.e. people living in local communities). Each of these entities can play a crucial role both during and in the aftermath of a natural disaster. Therefore, they all can benefit from disaster-preparedness planning. However, research shows that in disaster-preparedness planning, the roles and functions of the local communities are often not taken into consideration or are given lower priority.^3^ The extent to which disaster-preparedness programs fall short in engaging communities in pre-quake preparedness activities results in an increase in the local community’s likelihood of suffering from physical, financial, and emotional injuries, post-quake.^4^ Therefore, in providing effective disaster-preparedness programs, planning should be based on a periodic needs-assessment of the communities at risk. This will allow planners to consider knowledge, attitudes, behaviors, and skills of the at-risk communities in preparedness goals, designs, activities, and expected outcomes.

During any natural disaster, the vulnerable and affected communities are the center of considerable attention.^5^ For example, in the first hours after a natural disaster, due to the delay in transferring vital information; interruption in electronic communications; difficulties in accessing the affected areas; and organizing and mobilizing the aid forces, a considerable amount of time is wasted before the mobilized forces can reach the affected areas. At this time, commonly referred to as the “golden hours”, the survivors and the local residents are among the first who can help the injured effectively.^6^ 

Community-based disaster-preparedness programs can play a major role in training people to facilitate mobilization of aid across the affected areas. These programs should strive to deliver training materials that are in tune with the needs of the community members and are culturally sensitive and appropriate in content and design. Also, training in preparedness activities should be sensitive to the participants’ socio-demographic characteristics as well as their religious affiliations. Hence, identification of the means by which, and the mechanisms through which, culturally sensitive and appropriate training materials and activities can be delivered to the people in the communities are important steps in designing an effective community-based disaster-preparedness program.

Therefore, the specific aim of this study was to assess knowledge and attitudes of a sample of residents in Tehran in respect to what they consider to be an appropriate means and strategies for designing and delivering community-base earthquake-preparedness programs.

## Methods

**Sample and procedure**

This was a cross-sectional survey study conducted in 22 municipal districts in Tehran, the capital city of Iran. Participation in the study was voluntary and providing written consent and being 15≥ years old were the only inclusion criteria for the eligibility in the study. The study sample was interviewed over a period of four weeks. Cluster sampling was used in which the clusters were drawn systematically. A semi-structured questionnaire was developed by the members of the research team and was pilot-tested before its full implementation. Consented participants were interviewed by trained study interviewers on the door-step of their homes. The questionnaire included items related to knowledge and attitude of participants concerning issues related to earthquake-preparedness-training programs.

**Measures**

For the purpose of the current study, participants’ responses to the following questions were selected and described, from the main study questionnaire.

1.What measures should authorities or governmental organizations and agencies take to prevent earthquake-related causalities, assuming that eventually there will be an earthquake in Iran? Respondents were asked to prioritize their responses based on the following response categories: a) they should enforce the building-contractors’ compliance with the construction codes and regulations; b) they should increase manufacturing and use of earthquake-resistant buildings supplies; c) they should increase communities’ earthquake preparedness knowledge and awareness by means of mass media communication; d) they should provide regular earthquake-preparedness training opportunities for the members of the communities to participate and practice test drills; and e) they should assist members of the community to identify safe locations at home, in the neighborhood, and at work to be used as shelters in the event of any earthquake.

2.What types of media and what kinds of training and educational materials would you prefer to use to prepare yourself for an earthquake? Respondents were asked to prioritize their responses based on the following response categories: a) TV and radio; b) newspapers and magazines; c) flyers and posters; d) training workshops/classes; e) pamphlets; and f) videos, films, and /or an internet.

3.During the past 12 months, have you heard any “gossip” or “news” about the possibility of an upcoming earthquake in Iran? (Yes/No).

4.What was the source of this information? Response categories included: a) general public; b) TV; c) newsletters and magazines; d) radio; e) internet; f) academic institutions like universities and research institutes.

5.Which means of communications do you think are the most important for receiving news about the possibility of an earthquake?

6.What means of communications do you think are the most effective for receiving news/information during the aftermath of an earthquake?

7.Other than seismologists, who else do you think can correctly predict the occurrence of an earthquake?

8.During the aftermath of an earthquake, how can people access or obtain necessary information to properly deal with this disaster?

## Results

The respondents to the questionnaire included 1211 residents of Tehran comprising of 623 women (51/4%) and 588 men (48/6%). The age group for the men ranged from 15 to 100 years old (Mean= 37.3), and for the women it ranged from 15 to 84 (Mean= 34.5). The majority of the respondents had less than high school education (38.7%), and were married (60.9%) (Table 1).

**Table:** Table 1: **The overall sample characteristics (N=1211)**

		Number	Percent
Age group	15-20	216	17.8
	21-45	675	55.8
	46-65	264	21.8
	66 and above	56	4.6
Sex	Women	623	51.4
	Men	588	48.6
Education	Illiterate	55	4.5
	Under high school	469	38.7
	High school	375	31.0
	University degree	312	25.8
Marital status	Single	431	35.6
	Married	737	60.9
	Divorced, Widowed, etc	43	3.5
Profession	Jobless	104	8.6
	Housewife	336	27.7
	Labor, businessman	274	22.6
	Clerk, army	208	17.2
	University and school student	208	17.2
	Retired	81	6.7

Table 2 shows that “Enforcing the building-contractors’ compliance with the construction codes and regulations” was ranked as the first priority by 33.4% of the respondents. The lowest priority was given to “Assist members of the community to identify safe locations at home, in the neighborhood and at work to be used as shelters in the event of any earthquake”. This was chosen by 42.7% of the respondents.

**Table:** Table 2: **Respondents’ opinion on measures that authorities should undertake for earthquake- preparedness planning (N=1211)**

Priority measures	Ranks					
	1st	2nd	3rd	4th	5th
	F * (%)	F (%)	F (%)	F (%)	F (%)
1. Enforce the building contractors’ compliance with the construction codes and regulations	415	405	179	158	50
	(33.4%)	(31.6%)	(14.8)	(13/0)	(4.1%)
2. Increase manufacturing and use of earthquake resistance buildings supplies	324	409	194	132	147
	(26.8%)	(39.2%)	(16.0%)	(10.9%)	(12.1%)
3. Increase communities’ earthquake preparedness knowledge and awareness by means of mass media communication	252	177	382	228	192
	(20.8%)	(18.0%)	(31.5%)	(18.8%)	(15.9%)
4. Provide regular earthquake-preparedness training opportunities for the member of the communities to participate and practice test drills	104	143	164	296	304
	(8.7%)	(11.8%)	(13.5%)	(41.0%)	(25.2%)
5. Assist members of the community to identify safe locations at home, in the neighborhood, and at work to be used as shelters in the event of an earthquake.	125	78	292	199	517
	(10.3%)	(6.4%)	(24.2%)	(16.3%)	(42.7%)

* Frequency

**Figure3:**
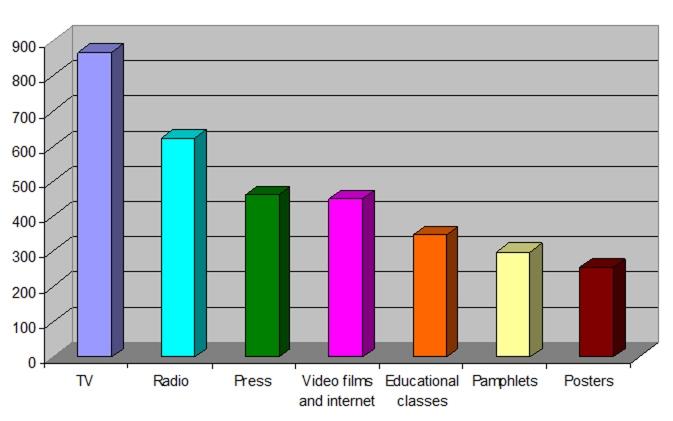
			Figure 3: People most voted media and tools for education and preparedness before earthquakes.

Table 3demonstrates the percentage of respondents who ranked their preferences for the types of media, training, and educational materials they prefer to use for earthquake preparedness purposes. Over 70% of the participants (71.7%) regarded TV as the most appropriate source of media communication to prepare people for an earthquake. This was followed by “radio” which was selected by 51.6% of respondents as the second most appropriate source of media communication. Instructional pamphlets (24.7%) and posters (21.1%) received the lowest priorities. (See also, Figure3).

**Table:** Table 3: **Respondents’ preferences for media and educational- preparedness materials (N=1211)**

Medium and tools	Ranks						
Priorities
	1st	2nd	3rd	4th	5th	6th	7th
	F * (%)	F * (%)	F * (%)	F * (%)	F * (%)	F * (%)	F * (%)
TV	868	173	78	39	38	7	8
	(71.7%)	(14.3%)	(6.4%)	(3.2%)	(3.1%)	(0.6%)	(0.7%)
Radio	35	624	215	113	76	99	49
	(2.9%)	(51.6%)	(17.8%)	(9.3%)	(6.3%)	(8.2%)	(4.0%)
Newspapers & magazines	40	138	462	163	124	109	175
	(3.3%)	(11.4%)	(38.2%)	(13.5%)	(10.2%)	(9.0%)	(14.5%)
Flyers & posters	53	32	112	256	216	228	314
	(4.4%)	(2.6%)	(9.2%)	(21.1%)	(17.8%)	(18.8%)	(25.9%)
Training workshops/classes	123	86	125	192	348	251	86
	(10.2%)	(7.1%)	(10.3%)	(15.9%)	(28.7%)	(20.7%)	(7.1%)
Pamphlets	46	87	109	267	276	299	127
	(3.6%)	(7.2%)	(9.0%)	(22.0%)	(22./8%)	(24.7%)	(10.5%)
Video, Films & Internet	47	70	110	184	133	217	450
	(3.9%)	(5.8%)	(9.1%)	(15.2%)	(11.0%)	(17.9%)	(37.2%)

* Frequency

The overwhelming majority of the respondents (95.1%) said they have heard either on the news or through some gossip that there would soon be an earthquake in the country. Table 4 presents the percentage of respondents who chose different sources from which they received this information. Approximately 80% of the participants picked “general public”; 38.9% TV; 25% newspapers and magazines; 8.9% radio; 6.7% internet and; 6.7% academic or research institutions.

**Table:** Table 4: **Sources from which participants received information about an upcoming earthquake**

Information Source	F	%
General public	915	78.9
TV	451	38.9
Newsletters and magazines	288	25.0
Radio	103	8.9
Internet	77	6.7
Universities and research institutes	77	6.7

When respondents were asked what means of communication they consider to be the most effective to deliver news/information about the aftermath of an earthquake, 70% endorsed news media in general. However, over fifty percent (58%) of the participants believed government officials and agencies are responsible for disseminating pertinent information about the possibility of an imminent earthquake. But 50.8% believed these agencies and organizations do not fulfill their responsibilities proficiently and effectively, and therefore, people are not provided with enough information in order to prepare them for an earthquake.

In addition to seismologists, respondents thought astronauts (32%), fortunetellers (32.3%), religious figures (34%), meteorologists (23%), and paleontologists (2%) could correctly predict the occurrence of any earthquake. Furthermore, 88.6% listed aid centers, mosques, newspapers and TV as the most important sources of information during the aftermath of an earthquake.

## Discussion

Several points can be deduced from the results of this study:

First, in any disaster-preparedness planning, it is important to take into account the local community’s input. This will allow programmers to recognize local communities’ needs and capacities, thereby matching training materials and activities in ways that would address pertinent needs of the communities.^7,8^ Earthquake-preparedness programs that are sensitive to the needs of the local community members can mobilize their efforts to develop their skills and capacities for dealing with any natural disasters.^9,10,11,12^

As long as communities are viewed as passive consumers of pre-packaged programs, often prepared by the experts, there will be less success in any health promotion activities or interventions.^13,14^ Although modern public health models offer a different perspective, they still assume individuals are rational beings and if provided with information they will behave rationally by avoiding risk and taking preventive actions.^13,14,15,16^ The authoritative nature of this model encounters much resistance from the communities to participate wholeheartedly in the innovative programs.^13,14,15,16,17,18^

Participatory theories and approaches, on the other hand, strive to take into account local communities’ culture and strengths, and build on their available assets while strengthening them by building a trusting relationship and mutual commitments.^19,20^ Participatory processes are not entirely new in disaster management.^19,20,21^ A participatory approach to earthquake-preparedness planning allows program planners to use methods, tools, media, and educational materials that are compatible with the culture, needs, and skills of the local communities.^20,21^

Second, the findings of this study, although preliminary, nonetheless reveal methods and tools that the local community considers to be most effective in earthquake-preparedness planning and management. As noted earlier, participants in this study believe that government organizations and agencies should take a more proactive role in ensuring the building contractors’ compliance with the construction codes. Also the overwhelming majority of the participants believed that TV and radio are the most appropriate sources of media communication to prepare them for an earthquake. Nearly everybody in the study believed that an eventual earthquake is inevitable (95%). Furthermore, approximately three-quarters of this sample considered the media as the source from which news about earthquake should be disseminated to the public.

Third, a community needs-assessment can inform the program planners, developers, and broadcasting organizations about where, what type of program, which day of the week, and what time of the day, earthquake-preparedness programs are most preferred to be implemented or broadcasted. Active participation of the local community in the design and production of earthquake-preparedness educational materials and programs helps the experts to customize their methods for the delivery of news, information, and educational materials in such a way that they represent the local people’s needs and situations. It will also empower the local community and help institutionalize such programs after the experts leave the community.

Fourth, regarding all aspects of earthquake preparedness management, and injury prevention, television with its wide coverage can be considered as an appropriate media for disseminating pertinent information. Cartoons, regularly scheduled programs and shows, as well as brief announcements are among various types of TV programming which could be used at many levels: national, regional, and local. In addition to TV, local radio stations should be equipped with sufficient financial and support systems to disseminate earthquake-related information to the communities where access to TV and internet is more limited (i.e. remote areas including rural communities). This is especially important, since TV broadcasting can be interrupted during an earthquake. Adding a weekly column in the most popular newspapers regarding earthquake preparedness and management would also be useful for the public. In addition, culturally sensitive disaster-preparedness videos films and CDs can reach various groups of society.

Fifth, the development of earthquake-resistance and a safe community requires a high level of collaboration between broadcasting organizations, seismologists, experts in the disaster-preparedness field, as well as the local community. This will allow for timely planning, development, and dissemination of essential information to all stakeholders including the local communities.

Last but not least, proper and timely process evaluation of the earthquake-preparedness programs can inform all stakeholders in charge of such programs of how communities are struggling with the initiation and sustainment of disaster preparedness programs. Long-term evaluation of these programs can highlight changes in the community’s knowledge, attitudes, behaviors, and skills related to disaster preparedness. It also can show the long-term effectiveness of setting up and equipping the local community to be in charge of, lead, and sustain such programs.

Although limited in design and analysis, we nevertheless hope that this study will inspire other researchers to further study and provide a better understanding of people’s perspectives on earthquake-preparedness planning. Such understanding is important for the development and monitoring of earthquake-preparedness programs and proper allocation of available resources.
